# Fast Analysis of Superoxide Dismutase (SOD) Activity in Barley Leaves Using Visible and Near Infrared Spectroscopy

**DOI:** 10.3390/s120810871

**Published:** 2012-08-07

**Authors:** Wenwen Kong, Yun Zhao, Fei Liu, Yong He, Tian Tian, Weijun Zhou

**Affiliations:** 1 College of Biosystems Engineering and Food Science, Zhejiang University, Hangzhou 310058, China; E-Mails: zjukww@163.com (W.K.); zy_super0201@163.com (Y.Z.); fliu@zju.edu.cn (F.L.); 2 School of Information and Electronic Engineering, Zhejiang University of Science and Technology, Hangzhou 310023, China; 3 College of Agriculture and Biotechnology, Zhejiang University, Hangzhou 310058, China; E-Mails: woshi.xiaot@163.com (T.T.); wjzhou@zju.edu.cn (W.Z.)

**Keywords:** visible and near infrared spectroscopy, barley, superoxide dismutase, variable selection, least squares-support vector machine, Gaussian process regression

## Abstract

Visible and near infrared (Vis/NIR) spectroscopy was investigated for the fast analysis of superoxide dismutase (SOD) activity in barley (*Hordeum vulgare* L.) leaves. Seven different spectra preprocessing methods were compared. Four regression methods were used for comparison of prediction performance, including partial least squares (PLS), multiple linear regression (MLR), least squares-support vector machine (LS-SVM) and Gaussian process regress (GPR). Successive projections algorithm (SPA) and regression coefficients (RC) were applied to select effective wavelengths (EWs) to develop more parsimonious models. The results indicated that Savitzky-Golay smoothing (SG) and multiplicative scatter correction (MSC) should be selected as the optimum preprocessing methods. The best prediction performance was achieved by the LV-LS-SVM model on SG spectra, and the correlation coefficients (*r*) and root mean square error of prediction (RMSEP) were 0.9064 and 0.5336, respectively. The conclusion was that Vis/NIR spectroscopy combined with multivariate analysis could be successfully applied for the fast estimation of SOD activity in barley leaves.

## 1. Introduction

Barley (*Hordeum vulgare* L.) is one of the most important cultivated crops in the World [1]. It is an ancient cereal grain, widely cultivated around the World, particularly in Asia and northern Africa. Barley is arguably the most widely adapted cereal grain species with production at higher latitudes and altitudes and farther into deserts than any other cereal crop [2,3]. Oxidative stress, resulting from the deleterious effects of reduced oxygen species, is an important phenomenon in many biological systems. Superoxide dismutase (SOD) is one of the protective enzymes. It has been identified as an essential component in organisms' defense mechanisms. In plants, SOD plays an important role in protecting against environmental adversity, as it can remove the free-radicals caused by environmental adversity and improve stress tolerance [4,5]. Nowadays the most popular method to test the activity of SOD is measuring its ability to inhibit the photochemical reduction of nitroblue tetrazolium. Traditional methods for detecting activity of SOD are laborious and time consuming. The traditional methods require destruction of the plants for SOD detection, which prevents the further use of the leaf samples. Moreover, these methods are not environmentally-friendly, because of the consumption of chemical reagents.

Near infrared (NIR) spectroscopy is a well-established technical for both quantitative and qualitative analysis in the field of agriculture [6,7]. As a quantitative analysis method, in this paper the prediction performance of Vis/NIR models was evaluated by the normally used correlation coefficients and root mean squares error of prediction. Normally, the correlation coefficients should be over 0.8 in the final prediction model for agriculture applications, which means the model could be considered as an effective and quantitative determination. To some extent, the method would be considered as semi-quantitative method or not feasible for this application if the correlation coefficient was less than 0.8 in final prediction performance. Then other relevant information selection methods or effective calibration methods should be introduced to improve the prediction performance. Near infrared (NIR) spectroscopy has been applied to barley by several researchers with various degrees of success. Williams *et al.*, reported satisfactory results (*r*^2^ = 0.66–0.96) in correlating the NIR spectral data of ground wheat and barley with their amino acid concentrations [8]. There were also some studies on disease recognition and quality analysis of barley by NIR spectroscopic techniques [9,10]. However, the correlation between NIR data and SOD activity in barley leaves has not been studied in detail.

The objectives of this experiment were to study the feasibility of using NIR spectroscopy to predict the activity of SOD in barley leaves, and compare the performance of different spectral preprocessing methods, different effective selection methods and calibration methods (partial least squares, least squares-support vector machine and Gaussian process).

## 2. Material and Methods

### 2.1. Sample Preparation

The experiments were conducted at the farm of Zhejiang University, Hangzhou (30°10′N, 120°12′E), China, in the year 2010. A herbicide (ZJ0273) was used as stressor with five concentrations (0, 50, 100, 500 and 1,000 mg/L) being applied at the two-leaf stage. A total of 75 barley samples were collected during the growing period (after treatment for 5, 10 and 15 days). The total samples were randomly divided into two sets, 50 samples for calibration and the remaining 25 samples for validation. No single samples were used in both calibration set and validation set at the same time.

### 2.2. Data Acquisition and Pre-Treatment

NIR spectra of the barley leaves were obtained using a Handheld FieldSpec spectrometer (Analytical Spectral Device, Boulder, CO, USA). The wavelength region is from 325 nm to 1,075 nm and the resolution of the instrument is 1.5 nm. Sample spectra acquired by averaging three spectra of one sample. In this study, three software packages were employed, including ASD View Spec Pro, Unscrambler V9.8 (CAMO AS, Oslo, Norway) and MATLAB V7.0 (The Math Works, Natick, MA, USA). Spectral pretreatment was necessary, because this could remove the spectral baseline shift, noise and light scatter influence [11]. Six different processing methods were applied in this study, including Savitzky-Golay smoothing (SG), standard normal variate (SNV), multiplicative scatter correction (MSC), first-derivative (1-Der) second-derivative (2-Der) and de-trending. The performance was determined by the prediction results of partial least squares (PLS) models.

Leaf superoxide dismutase (SOD) activity was analyzed by the method of Dhindsa *et al.* [12] by measuring its ability to inhibit the photochemical reduction of nitro blue tetrazolium (NBT). The reaction mixture (2.725 mL) contained 50 mM phosphate buffer, pH 7.8, 26 mM methionine, 20 μM riboflavin, 750 μM NBT and 1 μM EDTA. After adding enzyme solution (25 μL) and distilled water (250 μL) the reaction was allowed to run 15 min under 4,000 lx light. The absorbance by the reaction mixture at 560 nm was read.

### 2.3. Selection of Effective Wavelengths

Successive projections algorithm (SPA) and Regression coefficient (RC) analysis were proposed as variable selection strategy in this work. SPA is a forward selection method which starts with one wavelength, then incorporates a new one at each iteration, until a specified number *N* of wavelengths is reached. In this work, we set the maximum number of variables to be selected at 30. The details of SPA can be found in the literature [13,14]. PLS analysis is a widely used kind of linear regression method. It can analyze data with strongly collinearity, noisy, and numerous *X*-variables. The regression coefficients calculated from the spectral data could calculate the response value *Y*-variables (activity of SOD in barley) from the *X*-variables (spectral data). The value of coefficients indicates the importance of variables for predicting *Y*-variable. Therefore, PLS analysis can be used to select the effective wavelengths (EWs) by regression coefficient (RC) analysis [14].

### 2.4. Multivariate Calibration Methods

Four regression methods: partial least squares (PLS), multiple linear regression (MLR), least squares-support vector machine (LS-SVM), and Gaussian process (GP) were used for comparison of prediction performance.

PLS was performed by the software Unscrambler V9.8. The latent variables (LVs) were used as the direct inputs of PLS models to develop a relationship between the spectral data and the SOD activity in barley leaves. The number of latent variables was selected using full cross-validation procedure on the training set. MLR was still complied by the software Unscrambler V9.8.

The free LS-SVM v1.5 toolbox was applied with MATLAB V.7.0 to develop the LS-SVM models. Input variables, kernel function and model parameters were three crucial elements for LS-SVM model [14,15]. In this study, latent variables (LVs) extracted from PLS model and the selected EWs by SPA and RC analysis with different preprocessing methods were used as the input variables. The radial basis function (RBF) was recommended as kernel function. The model parameters gamma (γ) and sigma^2^ (σ^2^) were determined by a two-step grid search technique.

Gaussian process regression (GPR) is a recently developed machine learning method which is successfully applied to resolve regression and classification problems. Gaussian processes (GPs) are non-parametric models where a priori Gaussian process is directly defined over function values. The details of Gaussian process regression could be found in the literature [16,17]. The calculation was performed using MATLAB V7.0.

The evaluation standards include correlation coefficients (*r*), root mean squares error of prediction (RMSEP), bias, slope and offset. In this paper, *r* and RMSEP were the key indicators. The good model should be with higher *r* value and lower RMSEP, absolute bias and offset values, and the slope of the regression line should be closer to 1.

## 3. Results and Discussion

### 3.1. Full-Spectrum PLS Models

**Figure 1.**
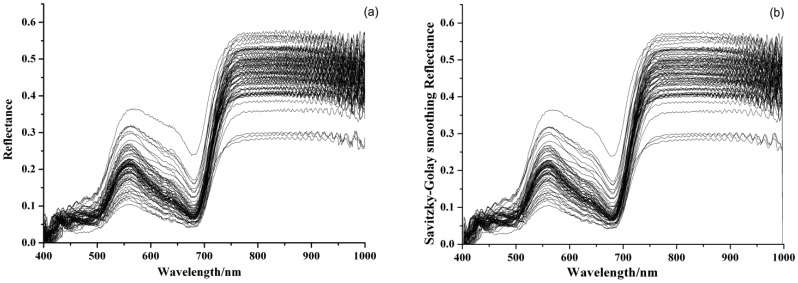

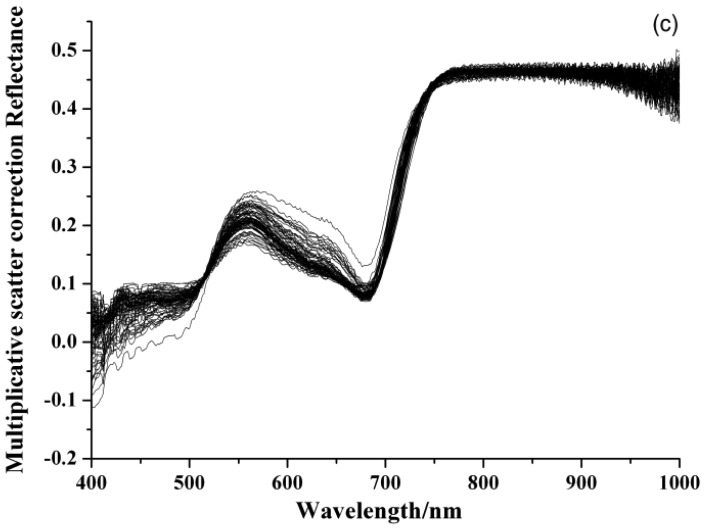
(**a**) Original spectra of Barley Leaves; preprocessed spectra by (**b**) Savitzky-Golay Smoothing (SG); (**c**) Multiplicative Scatter Correction (MSC).

Figure 1(a) shows the original visible/near infrared reflectance spectra of 75 barley leaves. The processed spectra by SG and MSC were shown in Figure 1(b,c). As can be seen, the trends of all samples were quite similar. There were a significant reflectance peak around 550 nm and an absorbance peak around 680 nm. This was caused by chlorophyll and showed a typical green plant spectral curve. The MSC preprocessed spectra in Figure 1(c) removed the baseline shift and improved the reproducibility. The statistical values of activity of SOD in barley leaves are shown in Table 1.

Seven different PLS models with full-spectrum were developed to evaluate the effects of different preprocessing methods. As mentioned above, the correlation coefficients (*r*) and root mean squares error of prediction (RMSEP) were adopted as key indicators. Table 2 shows the prediction results by the PLS models. The optimal PLS model was achieved by SG spectra with *r* = 0.8301 and RMSEP = 0.7060. Two preprocessing methods, SG and MSC were selected as optimized methods for further treatment. As shown in Table 2, the prediction results were not good enough as a quantitative analysis. The reason might be that the full-spectral region (601 variables) contained some redundant information, which impaired the performance of PLS model. Hence, it was necessary to bring in relevant variable selection methods (SPA and RC), and other calibration methods (LS-SVM and Gaussian process regression) to improve the prediction performance.

### 3.2. Selected EWs

**Figure 2.**
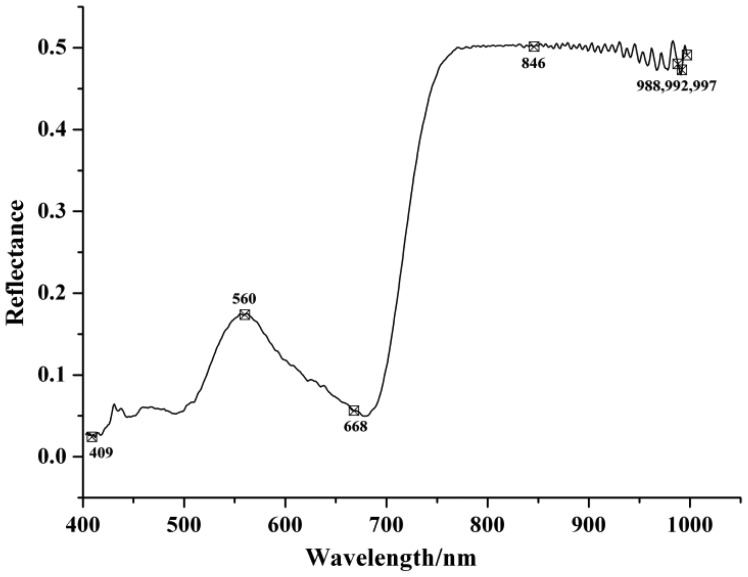
Selected EWs by SPA according to SG spectra.

**Figure 3.**
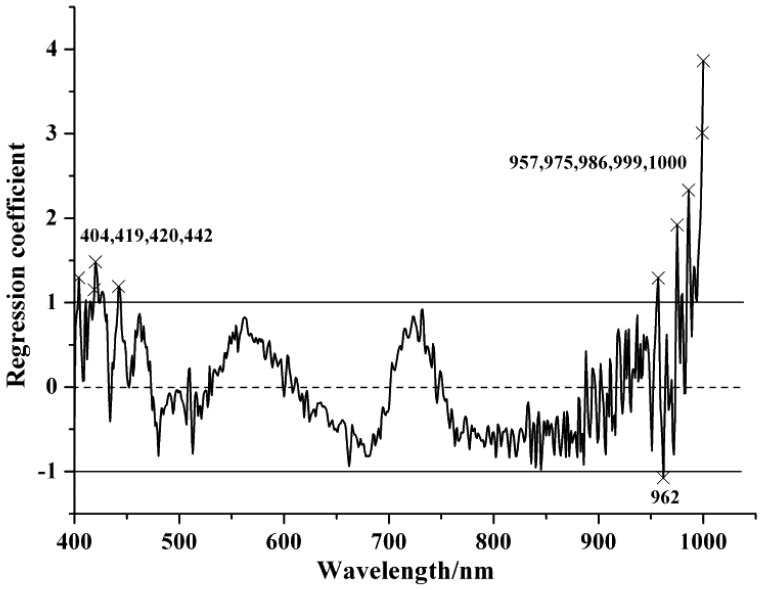
The regression coefficients of PLS.

Table 3 shows the selected EWs by SPA and RC with the optimized preprocessing spectra. In the SPA, the maximum number of selected variable was set as 30 and cross-validation was applied. In SPA, the cross-validation was carried out to the training set (calibration set) to make sure that the selection of relevant variables were stable and robust, and avoiding the possible over-fitting problems. The EWs selected by SPA were ranked in the order of importance in Table 3. The locations of the selected EWs by SPA according to SG spectra were shown in Figure 2 and the regression coefficient plot is shown in Figure 3.

The wavelengths selected between 973 and 1,020 nm (975, 981, 982, 984, 986, 992, 997, 999 and 1,000 nm) could be attributed to the second overtone of N-H stretching vibration. This region was considered as one of the characteristic bands of protein [18].

### 3.3. Comparison of Calibration Models with Simplified Input Variables

**Figure 4.**
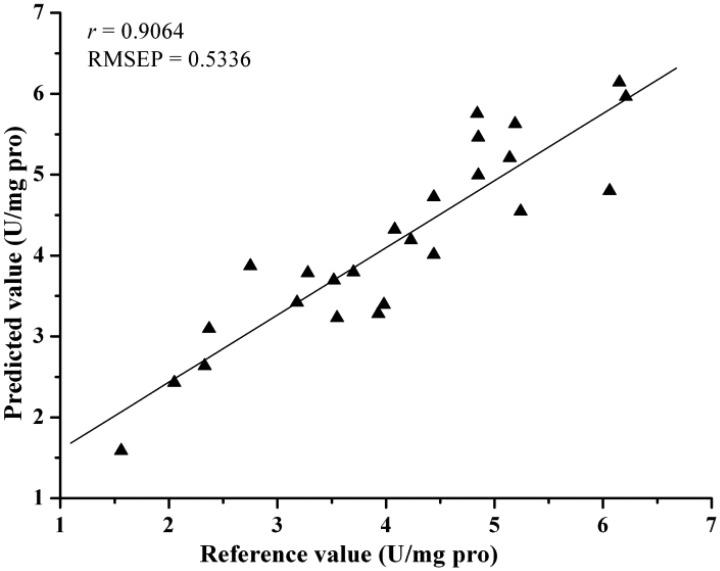
Predicted *vs.* reference activity of SOD by LV-LS-SVM (SG) in prediction set.

**Figure 5.**
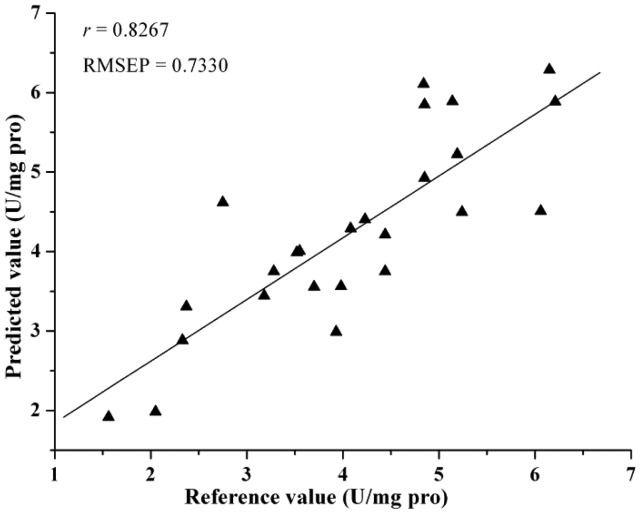
Predicted *vs.* reference activity of SOD by SPA-LS-SVM (SG) in prediction set.

Comparing the eight different models, they all achieved acceptable results. Table 4 lists the prediction results by different models. The best prediction performance was achieved by the LV-LS-SVM model with SG spectra, and correlation coefficients (*r*) = 0.9064 and root mean square error of prediction (RMSEP) = 0.5336. The LS-SVM and GPR models gave better prediction results than PLS and MLR models, which indicated that nonlinear calibration methods were more suitable for predicting activity of SOD in barley leaves. In this study, the performance of EWs selected by SPA was better than regression coefficients analysis. The possible reason was that SPA selected the relevant variables with least collinearity. However, both SPA and RC were considered useful methods for the selection of EWs, they selected just 2%–3% of the number of wavelengths as input for calibration models and gave acceptable results. The least EWs was 7, which selected by SPA according to SG spectra and the SPA-LS-SVM model gave the optimal result with *r* = 0.8267 and RMSEP = 0.7330, it was important for instrument development. The scatter plot for prediction sets by LV-LS-SVM(SG) and SPA-LS-SVM(SG) were shown in Figures 4 and 5.

## 4. Conclusions

Vis/NIR spectroscopy combined with multivariate analysis was successfully applied for the fast estimation of SOD activity in barley leaves. SG and MSC were selected as optimized processing methods by PLS. SPA and RC were successfully applied to select the most relevant EWs. Gaussian process regression gave good performance in this study, which indicated that it was a useful calibration method for the NIR spectroscopic technique. The best prediction performance was achieved by the LV-LS-SVM model with SG spectra, whereby the correlation coefficient (*r*) and root mean square error of prediction (RMSEP) were 0.9064 and 0.5336, respectively. In order to get a more stable prediction model and achieve fast detection in the field, further studies would be focused on increasing the number of samples, expanding the research spectra region and variable selection methods. Furthermore, comparison of prediction results under different environmental adversity is also important for further study.

## Figures and Tables

**Table 1.** Statistical values of activity of SOD in Barley Leaves (U/mg pro).

**Data set**	**Sample No.**	**Range**	**Mean**	**Standard deviation**
**Cal.**	50	1.52–6.43	4.13	1.342
**Val.**	25	1.56–6.21	4.08	1.273

**Table 2.** The prediction results of activity of SOD by PLS models with full-spectrum.

**Pretreatment**	**LV**	***r***	**RMSEP**	**Bias**	**Slope**	**Offset**
**Raw**	4	0.7742	0.8028	0.0816	0.6729	1.4151
**SG**	4	0.8301	0.7060	0.0784	0.7481	1.1052
**SNV**	3	0.8156	0.7258	−0.0421	0.7038	1.1654
**MSC**	5	0.8233	0.7179	−0.0384	0.7508	0.9775
**1-Der**	13	0.7943	0.7718	−0.1123	0.6886	1.1573
**2-Der**	3	0.5939	1.0417	−0.2265	0.4290	2.1013
**De-trending**	5	0.7629	0.8524	0.0178	0.7497	1.0383

**Table 3.** Selected EWs by SPA and RC.

**Methods**	**Pretreatment**	**No.**	**Selected EWs/nm**
SPA	Raw	18	453, 480, 970, 954, 408, 447, 469, 400, 1,000, 559, 497, 992, 406, 982, 404, 462, 434, 409
	SG	7	846, 997, 992, 560, 988, 409, 668
	MSC	10	869, 913, 984, 864, 749, 951, 854, 888, 918, 908
RC	Raw	10	404, 419, 420, 442, 957, 975, 986, 999, 1,000, 962
	SG	9	403, 419, 420, 443, 462, 957, 975, 986, 997
	MSC	15	400, 412, 434, 442, 681, 716, 723, 731, 864, 912, 947, 954, 965, 981, 1,000

**Table 4.** The prediction results by different models with optimal pretreatment.

**Models**	**Pretreatment**	**LV/EW/**(***γ*, σ^2^**)	**Validation**	

			***R****_v_*	**RMSEP**
SPA-PLS	Raw	12/18/-	0.6165	1.1324
	SG	7/7/-	0.7539	0.8627
RC-PLS	Raw	2/10/-	0.7035	0.8905
	MSC	4/15/-	0.6927	0.9416
SPA-MLR	Raw	-/18/-	0.6489	1.1280
	SG	-/7/-	0.7539	0.8627
LV-LS-SVM	Raw	6/-/(97.98,326.27)	0.8988	0.5521
	SG	6/-/(10.21,55.56)	0.9064	0.5336
SPA-LS-SVM	Raw	-/18/(2.10 × 10^3^,562.91)	0.7203	0.9293
	SG	-/7/(361.12,129.21)	0.8267	0.7330
RC-LS-SVM	Raw	-/10/(3.14,29.90)	0.7798	0.7838
	SG	-/9/(8.61,78.30)	0.7798	0.7838
SPA-GPR	Raw	-/18/-	0.4771	1.1380
	SG	-/7/-	0.8200	0.7377
RC-GPR	Raw	-/10/-	0.7840	0.7776
	SG	-/9/-	0.7440	0.8326
